# Loss of *G_q/11_* Genes Does Not Abolish Melanopsin Phototransduction

**DOI:** 10.1371/journal.pone.0098356

**Published:** 2014-05-28

**Authors:** Kylie S. Chew, Tiffany M. Schmidt, Alan C. Rupp, Paulo Kofuji, Jeffrey M. Trimarchi

**Affiliations:** 1 Department of Biology, Johns Hopkins University, Baltimore, Maryland, United States of America; 2 Department of Neuroscience, University of Minnesota, Minneapolis, Minnesota, United States of America; 3 Department of Genetics, Development and Cell Biology, Iowa State University, Ames, Iowa, United States of America; Virginia Tech Carilion Research Institute, United States of America

## Abstract

In mammals, a subset of retinal ganglion cells (RGCs) expresses the photopigment melanopsin, which renders them intrinsically photosensitive (ipRGCs). These ipRGCs mediate various non-image-forming visual functions such as circadian photoentrainment and the pupillary light reflex (PLR). Melanopsin phototransduction begins with activation of a heterotrimeric G protein of unknown identity. Several studies of melanopsin phototransduction have implicated a G-protein of the G_q/11_ family, which consists of *Gna11*, *Gna14*, *Gnaq* and *Gna15*, in melanopsin-evoked depolarization. However, the exact identity of the G_q/11_ gene involved in this process has remained elusive. Additionally, whether G_q/11_ G-proteins are necessary for melanopsin phototransduction *in vivo* has not yet been examined. We show here that the majority of ipRGCs express both *Gna11* and *Gna14*, but neither *Gnaq* nor *Gna15*. Animals lacking the melanopsin protein have well-characterized deficits in the PLR and circadian behaviors, and we therefore examined these non-imaging forming visual functions in a variety of single and double mutants for *G_q/11_* family members. All *G_q/11_* mutant animals exhibited PLR and circadian behaviors indistinguishable from WT. In addition, we show persistence of ipRGC light-evoked responses in *Gna11^−/−^; Gna14^−/−^* retinas using multielectrode array recordings. These results demonstrate that G_q_, G_11_, G_14_, or G_15_ alone or in combination are not necessary for melanopsin-based phototransduction, and suggest that ipRGCs may be able to utilize a G_q/11_-independent phototransduction cascade *in vivo*.

## Introduction

Intrinsically photosensitive retinal ganglion cells (ipRGCs) comprise a distinct subset of retinal ganglion cells (RGCs) and express the photopigment melanopsin (*Opn4*) [1]. ipRGCs constitute the sole conduit of light information from the retina to non-image forming visual centers in the brain and are responsible for driving a variety of behaviors [2], [3]. These behaviors include circadian photoentrainment, which is the process by which the circadian clock is aligned to the environmental light-dark cycle, and the pupillary light reflex (PLR), in which the area of the pupil changes in response to changes in light intensity.

Despite the well-established role for ipRGCs and melanopsin in the regulation of non-image forming visual functions, little is known about the molecular components of melanopsin phototransduction. Previous research has suggested that ipRGCs likely utilize a phototransduction pathway similar to that used in *Drosophila* microvillar photoreceptors [1], [4], in which the activated opsin stimulates a G_q/11_ protein. In *Drosophila*, the α-subunit of the G_q/11_ protein activates phospholipase C-β (PLC-β), which results in the opening of TRP and TRPL channels allowing Na^+^ and Ca^2+^ to flow into the cell resulting in depolarization of the rhabdomere in response to light [5], [6].

Homologs of the components of the *Drosophila* phototransduction pathway are found in mice. Specifically, there are four *G_q/11_* genes (*Gnaq*, *Gna11*, *Gna14*, and *Gna15*), four *Plc-β* genes (*Plc-β1* – *4*), and seven *Trpc* channel genes (*Trpc1*-*7*). The tandemly duplicated *Gna15* and *Gna11* genes are linked to mouse chromosome 10 [7], [8], and *Gnaq* and *Gna14* colocalize to mouse chromosome 19 [9]. To date, there have been several electrophysiological studies implicating *G_q/11_*, *Plc-β*, and *TrpC* genes in ipRGC phototransduction [4], [10], [11]. However, there have been no functional studies investigating the identity of the G_q/11_ protein utilized by melanopsin *in vivo* or any studies of the effects of the loss of any presumptive ipRGC phototransduction genes on behavior. In this study, we sought to determine the identity(ies) of the G_q/11_ protein(s) utilized for melanopsin phototransduction *in vivo*.

We performed single-cell RT-PCR on individual ipRGCs to determine which of the genes were expressed in ipRGCs and if there was heterogeneity in their expression among the ipRGC population. Similar to previous studies, we found that the majority of ipRGCs express both *Gna11* and *Gna14*, but not *Gnaq* or *Gna15*. Since loss of the melanopsin protein results in well-characterized deficits in the pupillary light reflex and circadian behaviors, we examined these non-imaging forming visual functions in *Gna11^−/−^; Gna14^−/−^* (*Gna11*; *Gna14* DKO) mice and *Gnaq^flx/flx^*; *Gna11^−/−^*; *Opn4^Cre/+^* (*Gnaq*; *Gna11* DKO) mice as well as several single *G_q/11_* gene knockouts [9], [12]–[14]. All genotypes examined exhibited non-image forming visual functions indistinguishable from WT. Furthermore, multielectrode array recordings revealed no deficits in ipRGC light responses in *Gna11*; *Gna14* DKO animals. Contrary to previous work, this study indicates that ipRGCs may be able to utilize a G_q/11_-independent phototransduction cascade *in vivo*.

## Results

### *Gna11* and *Gna14* are expressed in ipRGCs

**Figure 1:**
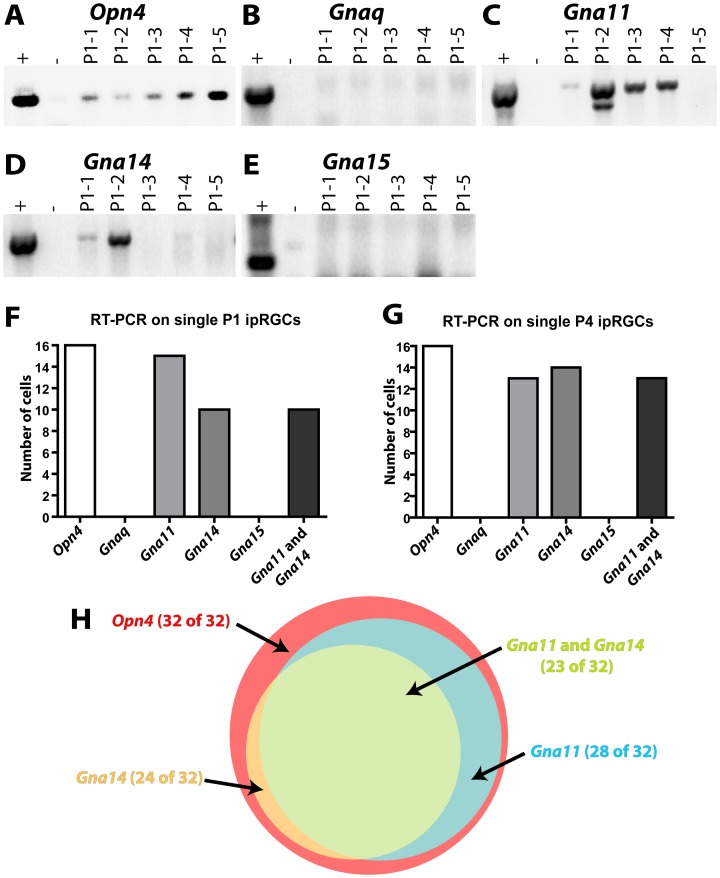
*Gna11* and *Gna14* are expressed in ipRGCs, often in combination. **A–E**. Representative images of RT-PCR analysis of single ipRGCs for *Opn4*, *Gnaq*, *Gna11*, *Gna14*, and *Gna15*. All representative gels show RT-PCR analysis of single ipRGCs taken from P1 *Opn4^Cre/+^*; *Z/EG* mice. Each lane represents one cell, the positive control is whole retinal RNA, and the negative control is water. **F–G** Summary of expression of G_q/11_ family members in the 16 ipRGCs obtained from P1 and P4 *Opn4^Cre/+^*; *Z/EG* mice. All cells expressed melanopsin. 15 cells expressed *Gna11*, 10 of which also expressed *Gna14*. **H**. Venn diagram showing the distribution of G_q/11_ family member expression in all 32 ipRGCs sampled.

Previous reports have shown that *G_q/11_* genes are expressed in ipRGCs. However, there has been disagreement regarding which *G_q/11_* genes are actually expressed, with one study reporting heterogeneous expression of each of the four *G_q/11_* genes and another reporting primarily *Gna14* and some *Gna11* expression [4], [15]. We therefore sought to definitively identify which *G_q/11_* genes are expressed in ipRGCs. We isolated individual ipRGCs by dissociating retinas of *Opn4^Cre/+^ Z/EG* mice, in which ipRGCs ipRGCs are labeled with GFP, and picking individual ipRGCs with a microneedle. We specifically chose to utilize retinas from P1 and P4 mice since there is GFP labeling of some cones in adult *Opn4^Cre/+^ Z/EG* mice [16]. By RT-PCR, we confirmed that the 32 isolated cells expressed melanopsin (Figure 1A, F–H), and then screened those 32 melanopsin-expressing cells for the four *G_q/11_* genes (Figure 1B–H). 23 of the 32 ipRGCs expressed both *Gna11* and *Gna14*, and an additional 6 cells expressed either *Gna11* or *Gna14* (Figure 1F–H). Neither *Gnaq* nor *Gna15* were detected in any of the melanopsin-expressing cells, and 3 melanopsin-expressing cells had no detectable levels of any *G_q/11_* gene (Figure 1F–H).

### Loss of *G_q/11_* genes does not affect non-image forming visual functions

**Figure 2:**
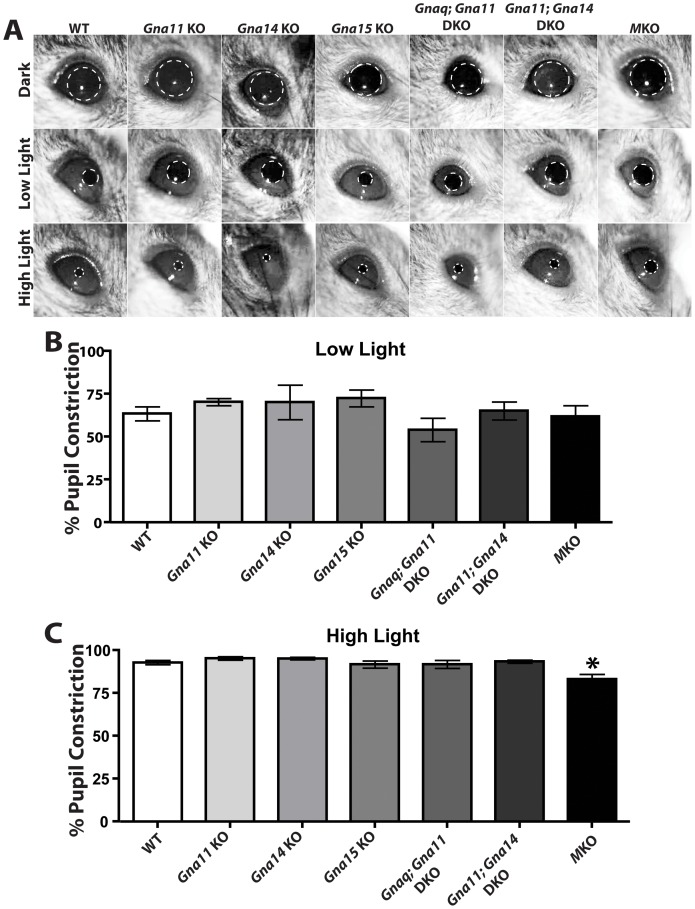
G_q/11_ mutant lines exhibit pupillary light reflex indistinguishable from WT. **A**. Representative images of the pupil constriction in WT (16 animals), *Opn4^LacZ/LacZ^* (MKO, 7 animals), *Gna11^−/−^* (*Gna11* KO, 4 animals), *Gna14^−/−^* (*Gna14* KO, 5 animals), *Gna15^−/−^* (*Gna15* KO, 7 animals), *Gnaq^flx/flx^*; *Gna11^−/−^*; *Opn4^Cre/+^* (*Gnaq; Gna11* DKO, 9 animals), and *Gna11^−/−^; Gna14^−/−^* (*Gna11; Gna14* DKO, 7 animals) at both high (1.4×10^16^ photons/cm^2^/sec) and low (7.3×10^13^ photons/cm^2^/sec) light intensities. **B–C**. Quantification of the pupillary light reflex at low (7.3×10^13^ photons/cm^2^/sec) and high (1.4×10^16^ photons/cm^2^/sec) light intensities. All animals exhibited pupillary light reflex indistinguishable from WT. One-way ANOVA with Tukey post-hoc analysis. Error bars represent s.e.m.

Mice that lack melanopsin phototransduction due to loss of melanopsin have several well characterized deficits in non-image forming visual behaviors including defects in the PLR at high light intensities and a deficit in circadian period lengthening in response to constant light. Since *Gna11* and *Gna14* were the only *G_q/11_* genes identified as being expressed in ipRGCs and nearly all cells tested expressed at least one, we produced *Gna11^−/−^; Gna14^−/−^* (*Gna11; Gna14* DKO) mice from previously published single knockouts [14], [17], [18]. We recorded the pupillary light reflex of 4–6 month old WT (n = 16), *Opn4^LacZ/LacZ^* (MKO; n = , 7), *Gna11^−/−^* (*Gna11* KO; n = 4), *Gna14^−/−^* (*Gna14* KO; n = 5), *Gna15^−/−^* (*Gna15* KO; n = 7), *Gnaq^flx/flx^*; *Gna11^−/−^*; *Opn4^Cre/+^* (*Gnaq; Gna11* DKO; n = 9), and *Gna11^−/−^; Gna14^−/−^* (*Gna11; Gna14* DKO; n = 7) at both low and high light intensities (Figure 2). Consistent with previous studies [19], MKOs exhibited deficits at high light intensities. Surprisingly, all mice mutant for *G_q/11_* genes were indistinguishable from WT animals at both low and high light intensities (Figure 2).

**Figure 3:**
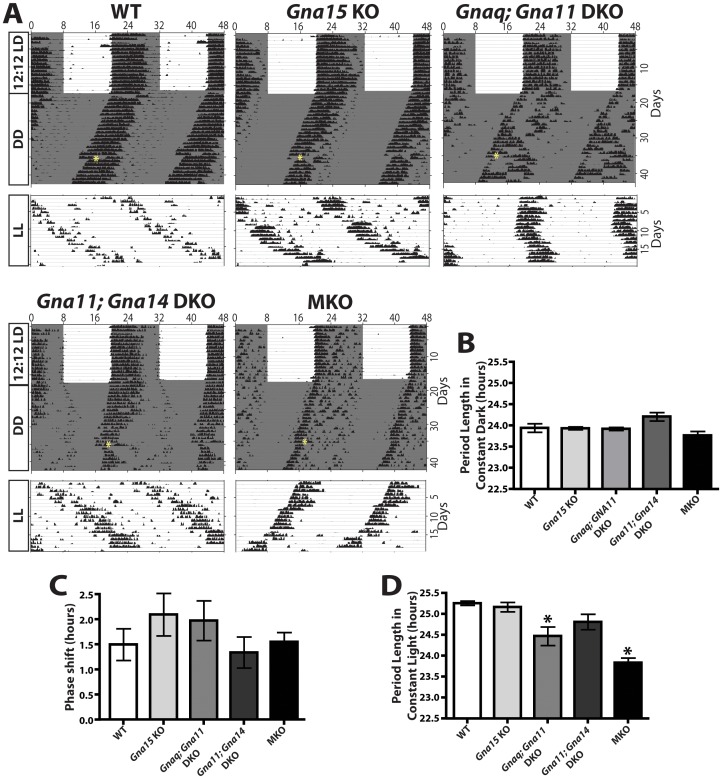
G_q/11_ mutant lines exhibit circadian behaviors indistinguishable from WT. **A**. Representative actograms of wheel running activity from WT (14 animals), MKO (9 animals), *Gna15* KO (7 animals), *Gnaq; Gna11* DKO (8 animals), and *Gna11; Gna14* DKO (7 animals) mice under a 12∶12 LD cycle, constant darkness, and constant light. The white background indicates light, grey background indicates darkness, and the yellow asterisk indicates a 15-minute light pulse at circadian time (CT) 15. All mice photoentrained to the LD cycle. **B**. Quantification of free-running period under constant dark conditions. All animals exhibited circadian periods indistinguishable from WT. One-way ANOVA with Tukey post-hoc analysis. Error bars represent s.e.m. **C**. Quantification of phase shifting to a 15-minute light pulse given at CT 15. All animals exhibited phase shifting indistinguishable from WT. One-way ANOVA with Tukey post-hoc analysis. Error bars represent s.e.m. **D**. Quantification of free running period under constant light. As previously reported, MKO mice exhibited reduced lengthening of their circadian period under constant light conditions. *Gnaq*; *Gna11* DKO exhibited a slight reduction in the lengthening of their circadian period in constant light, and their period length was significantly different from both WT and MKO. *Gna15* KO and *Gna11; Gna14* DKO exhibited lengthened periods that were indistinguishable from WT. One-way ANOVA with Tukey post-hoc analysis. Error bars represent s.e.m.

We also recorded wheel-running activity in 4–6 month old WT (n = 14), MKO (n = 9), *Gna15* KO (n = 7), *Gnaq; Gna11* DKO (n = 8), and *Gna11; Gna14* DKO (n = 7) mice to measure the daily activity rhythms of these mice (Figure 3). We conducted these measurements under three different conditions: a 12∶12 light/dark cycle, constant darkness, and constant light. We also administered a 15-minute light pulse in constant darkness to determine the amplitude of the light-evoked circadian phase shifts in each mouse line. All genotypes were able to photoentrain to the 12∶12 light/dark cycle. All mutant lines exhibited a normal circadian period under constant darkness (WT: 23.85±0.36 hours, MKO: 23.68±0.26 hours, *Gna15* KO: 23.84±0.08 hours, *Gnaq; Gna11* DKO: 23.83±0.10 hours, and *Gna11; Gna14* DKO: 24.01±0.24 hours) (Figure 3A, B). All mice phase shifted normally to a light pulse presented at CT15. We observed no deficits in phase delay among any genotypes tested (WT: 1.40±0.78 hours, MKO: 1.45±0.49 hours, *Gna15* KO: 1.96±1.04 hours, *Gnaq; Gna11* DKO: 1.85±0.74 hours, and *Gna11; Gna14* DKO: 1.25±0.65 hours) (Figure 3A,C). Melanopsin knockout animals have well-characterized deficits in circadian period lengthening under constant light [20] that we confirmed here (WT period: 25.08±0.17 hours, MKO period: 23.75±0.28 hours) (Figure 3A, D). In contrast, *Gna15* KOs (24.99±0.28 hours) and *Gna11; Gna14* DKOs (24.66±0.42 hours) were indistinguishable from WT mice. While *Gnaq; Gna11* DKO animals (24.34±0.5 hours) did show a significantly shorter period than WT animals, the period was still significantly longer than MKO animals (Figure 3A, D).

### ipRGC light responses persist in *Gna11*; *Gna14* double knockouts

**Figure 4:**
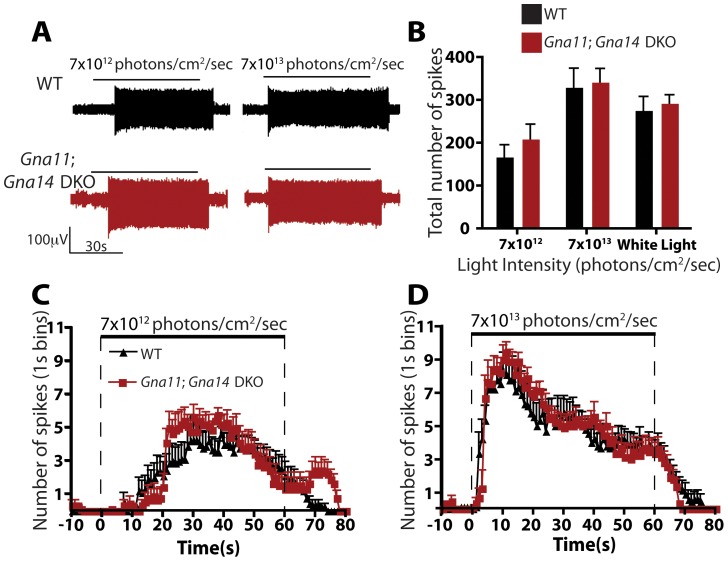
ipRGC intrinsic phototransduction persists in *Gna11*; *Gna14* DKO mice. **A**. Representative voltage traces for ipRGC intrinsic light responses in WT and *Gna11*; *Gna14* DKO retinas at two 480 nm light intensities (7×10^12^ and 7×10^13^ photons/cm^2^/sec). Horizontal bar represents light stimulation (60 sec). Vertical scale bar is 100 µV. **B**. Total number of spikes in ipRGCs light responses to two 480 nm light intensities (7×10^12^ and 7×10^13^ photons/cm^2^/sec) and white light (267 mW/cm^2^). ipRGC light responses in *Gna11*; *Gna14* DKO were indistinguishable from WT. Student's t-test. Error bars represent s.e.m. **C–D**. Quantification of the number of spikes, in 1 second bins, during a 60 second pulse of either 7×10^12^ photons/cm^2^/sec or 7×10^13^ photons/cm^2^/sec 480 nm light. Photoresponses in *Gna11*; *Gna14* DKO mice were indistinguishable from WT. Student's t-test. Error bars represent s.e.m.

The lack of behavioral deficits in *G_q/11_* mutant animals led us to examine whether melanopsin phototransduction is perturbed at the cellular level in these lines. We therefore examined the light responses of ipRGCs in isolated retinas of WT and *Gna11; Gna14* DKO mice using multielectrode array (MEA) recordings. We recorded from retinas of postnatal day 3 mice, since it has been shown that outer retinal signaling to ganglion cells is not present at early postnatal ages [21], and thus ipRGCs constitute the only light-responsive ganglion cells at this age [22], [23]. Nonetheless, to guarantee that all detected light responses were from ipRGCs, we included a cocktail of synaptic blockers in the Ames' medium to inhibit any glutamatergic, GABAergic, and glycinergic signaling to ipRGCs. Additionally, we included cholinergic blockers to minimize interference from retinal waves present at this developmental stage [24]. Retinas were dark adapted for at least 20 min and then stimulated with diffuse, uniform light of both low (7×10^12^ photons/cm^2^ · sec) and high light intensity (7×10^13^ photons/cm^2^ · sec) for 60 sec at 480 nm, the peak wavelength for melanopsin activation [25], [26]. We also stimulated the retinas with bright white light (267 mW/cm^2^). The retinas were allowed to readapt to dark for 5 min between stimulations. Figure 4A shows representative voltage traces of typical ipRGCs in WT and *Gna11; Gna14* DKO mice at both low and high light intensity. We found that *Gna11; Gna14* DKO ipRGCs were indistinguishable from the WT controls. ipRGCs in both WT and *Gna11; Gna14* DKO mice responded to increasing light intensities with increased spiking (Figure 4B) that reached maximum levels several seconds following light onset. After light offset, ipRGCs continued to spike for as long as 20 seconds (Figure 4A, C, D). These slow dynamics are consistent with previous descriptions of melanopsin-dependent light responses [23], [27]–[29]. These data show that despite the fact that *Gna11* and *Gna14* were the only *G_q/11_* genes expressed in ipRGCs, they are not required for melanopsin phototransduction.

### Other *G_q/11_* genes are up-regulated in single and double *G_q/11_* knockouts

**Figure 5:**
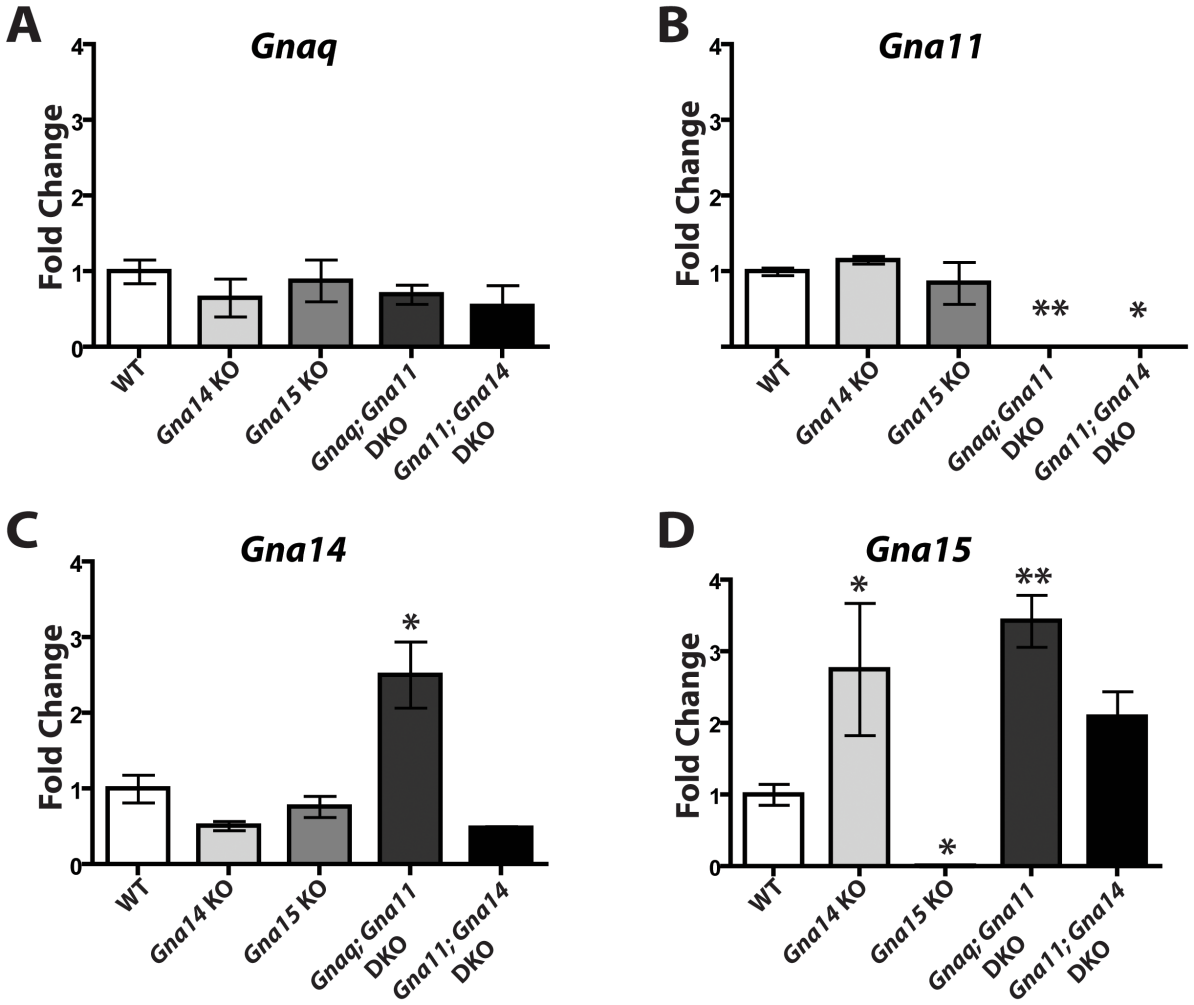
G_q/11_ family members are upregulated in the retinas of some G_q/11_ mutant lines. **A–D**. Expression levels of *Gnaq*, *Gna11*, *Gna14*, and *Gna15* in the retina relative to WT in *Gna14* KO, *Gna15* KO, *Gnaq; Gna11* DKO, and *Gna11; Gna14* DKO mice. Normalized to levels of 18S RNA. (N = 3 mice for each; 2 retinas per RNA sample). * indicates P<0.05 by one-way ANOVA with Tukey post-hoc analysis. Error bars represent s.e.m.

Since *Gna11* and *Gna14* were the only *G_q/11_* genes detected in ipRGCs, we were surprised that *Gna11; Gna14* DKO mice did not recapitulate any of the phenotypes observed in melanopsin knockout animals. To test whether removal of one or two *G_q/11_* genes results in upregulation of other *G_q/11_* family members, we performed quantitative RT-PCR on RNA extracted from the retinas of mutant mice (Figure 5). We measured the mRNA levels of *Gnaq*, *Gna11*, *Gna14*, and *Gna15* in WT, *Gna14* KO, *Gna15* KO, *Gnaq; Gna11* DKO, and *Gna11; Gna14* DKO mice. We found that all animals had levels of *Gnaq* mRNA that were indistinguishable from WT (Figure 5A). It is important to note that in *Gnaq*; *Gna11* DKOs, *Gnaq* is conditionally knocked-out in ipRGCs (*Gnaq^flx/flx^*; *Gna11^−/−^*; *Opn4^Cre/+^*); therefore, we did not expect a significant reduction in whole retinal *Gnaq* mRNA in these mutants. *Gna14* KOs and *Gna15* KOs exhibited normal levels of *Gna11* mRNA, while, *Gnaq; Gna11* DKOs, and *Gna11; Gna14* DKOs had undetectable levels (Figure 5B). Levels of *Gna14* mRNA were reduced in *Gna14* KOs and *Gna11; Gna14* DKOs, but increased in *Gnaq; Gna11* DKOs (Figure 5C). *Gna14* mRNA levels are not abolished in *Gna14* KOs and *Gna11; Gna14* DKOs because the *Gna14* KO line was created by knocking a neo cassette into exon 3 of the gene. This removes part of exon 3 and results in a frameshift. Thus, while, mRNA is still produced from the *Gna14* locus in *Gna14* KOs, it encodes a nonsense protein. Additionally, as indicated by the reduced mRNA levels, the mutant transcript is degraded. *Gna15* mRNA was undetectable in *Gna15* KOs, but increased in *Gna14* KOs, *Gnaq; Gna11* DKOs, and *Gna11; Gna14* DKOs (Figure 5D). These data indicate there is upregulation of other *G_q/11_* genes in some *G_q/11_* knockout lines; however, it remains unknown whether the upregulation occurs in ipRGCs and if such upregulation would be sufficient to drive melanopsin phototransduction.

## Discussion

In this study, we provide the first investigation of the melanopsin phototransduction pathway *in vivo*. We determined that genetic inactivation of the G_q/11_ proteins that are normally expressed in ipRGCs does not abolish melanopsin–dependent behaviors or electrophysiological responses. Specifically, we found that no tested *G_q/11_* knockout line exhibited the behavioral deficits observed in melanopsin knockout mice. All tested *G_q/11_* mutant lines exhibited circadian behaviors and pupillary light reflexes that were indistinguishable from WT mice. Additionally, using single-cell RT-PCR for *G_q/11_* genes in ipRGCs, we found only expression of *Gna11* and *Gna14*, often expressed together. However, using multielectrode array we detected no changes in intrinsic light responses of ipRGCs in *Gna11; Gna14* DKO compared to WT controls.

Previous reports have shown expression of *G_q/11_* genes in ipRGCs although there were inconsistencies as to which *G_q/11_* genes were detected [4], [15]. Specifically, in Graham et al. the authors used single cell RT-PCR and determined that expression of all four *G_q/11_* genes can be detected in ipRGCs, although expression was heterogeneous among the cells sampled and *Gna14* was detected in the majority of cells [4]. Siegert et al. examined ipRGCs as a population and reported expression of *Gna11* and *Gna14* [15], which is consistent with our findings here. However, neither of these studies investigated the function of ipRGCs in the absence of any of these specific genes and in fact Siegert et al. observed the expression of other heterotrimeric G proteins [15].

Electrophysiological investigations of ipRGC phototransduction have supported the involvement of the *G_q/11_* pathway. Specifically, Xue et al. showed that melanopsin phototransduction is substantially reduced in the absence of *Plc-β4* [10]. In agreement with work from Perez-Leighton et al., Xue and co workers additionally showed that loss of both *Trpc6* and *7* virtually abolished the melanopsin-dependent photoresponse suggesting that *Trpc6* and *7* function in a combinatorial fashion [10], [11]. Since G_q/11_ family members are defined based on their ability to activate PLC, it is reasonable to predict that if *Plc-β* is a critical component of melanopsin phototransduction then there must also be a member of G_q/11_ family involved. This prediction was supported with the use of pharmacological inhibitors of the G_q/11_ family on dissociated ipRGCs [4]. However, here, we show that *in vivo*, mice mutant for *G_q/11_* family members do not exhibit the behavioral deficits indicative of a loss of melanopsin-dependent light responses.

Several possibilities exist to explain these discrepancies. One is that G_q/11_ signaling is not required for melanopsin phototransduction. Siegert et al. observed expression of other heterotrimeric G proteins [15] and thus melanopsin could activate a G_i_ or G_o_ protein, as has been observed *in vitro* [30], the dissociation of which could result in the beta/gamma subunit activating PLC-β4 as has been observed with PLC-β1 and 3 [31]. Another possibility is that there is compensatory upregulation from other remaining G_q/11_ family members in the tested mutant lines. Our data supports this possibility since *Gna14* and *Gna15* were upregulated in *Gnaq; Gna11* DKOs. Also, *Gna15* was upregulated in *Gna14* knockouts; although, the increase in *Gna15* expression was not significant in *Gna11; Gna14* DKOs. However, our qRT-PCR experiments were performed on whole retinal RNA, and expression of *Gna15* has not consistently been reported in ipRGCs. Thus, it is unknown whether there is ectopic expression of *Gna15* in ipRGCs in *G_q/11_* knockout lines. Whether other G_q/11_ family members are upregulated in the conventional G_q_ knockout lines could be investigated by creating a mouse line that has all four *G_q/11_* genes knocked-out in ipRGCs. Due the fact that *G_q/11_* genes exist as two closely linked pairs on two single chromosomes, this quadruple knockout will require creation of a new mutant line in which the linked genes are knockout together. This mouse line would definitively reveal the contribution of the *G_q/11_* class alpha subunits to the melanopsin phototransduction cascade.

Additionally, it remains possible that *Gna11* and *Gna14* are required for the activation PLC-β4 and TRPC6/7, but this pathway is not required for normal ipRGC-mediated behavior. In support of this idea, a small residual light-activated current exist in *Plc-β4^−/−^* and *Trpc6/7^−/−^* ipRGCs [10]. Importantly, voltage recordings were not performed in these mutants. Therefore, it remains possible that this small residual current is sufficient to drive spiking in ipRGCs, which then drives normal non-image-forming visual behaviors. To test this, behavioral assays need to be performed on *Plc-β4^−/−^* and *Trpc6/7^−/−^* mice.

It is important to note that ipRGCs are not a homogeneous population and ipRGC subtypes (termed M1–M5) have stereotyped yet distinct electrophysiological light responses. Thus, it is possible there is variability in the components of the melanopsin phototransduction cascade among ipRGC subtypes. The study showing that ipRGCs have a severe reduction in their intrinsic light responses in mouse lines mutant for *Trpc6* and *-7* channel genes and *Plc-β4* [10] only examined the M1 ipRGC subtype, and in *Trpc6* mutant mice, both M1 and M2 ipRGCs show some deficits in melanopsin-dependent light responses [11]. While M1 ipRGCs are the predominant subtype mediating circadian behaviors, non-M1 ipRGCs may contribute to the PLR [16]. It remains unknown whether the intrinsic responses of other ipRGC subtypes are affected in *Trpc6* and *Trpc7* double knockouts or in *Plc-β4* knockouts. Because we picked single cells for RT-PCR at a developmental time, we could not be certain whether we were picking M1 or non-M1 ipRGCs. A careful analysis of the phototransduction in M1 versus non-M1 ipRGCs has interesting functional and evolutionary implications.

## Materials and Methods

### Ethics Statement

All protocols, animal housing, and treatment conditions were approved by the Johns Hopkins University Animal Care and Use Committee (IACUC).

### Animal Models

All mice were of a mixed background (C57BL/6;129SvJ). Melvin Simon at University of California San Diego generously provided *Gnaq^flx/flx^*; *Gna11^−/−^* animals and *Gna14^−/−^* animals [13], [14], [17], and Thomas Wilkie at University of Texas Southwestern generously provided *Gna15^−/−^* animals [12]. *Gnaq^flx/flx^*; *Gna11^−/−^* animals were crossed into our *Opn4^Cre/+^* line to produce *Opn4^Cre/+^*; *Gnaq^flx/flx^*; *Gna11^−/−^* mice. *Gnaq^flx/flx^*; *Gna11^−/−^* mice were also crossed with *Gna14^−/−^* mice to produce *Gna11^−/−^*; *Gna14^−/−^* animals.

### Single Cell RT-PCR

Single ipRGCs were isolated from *Opn4^Cre/+^*; *Z/EG* mice following the protocol described in [32]. Reverse transcription of the RNA from single cells from P1 and P4 retinas, and amplification of the cDNA was performed as described in [32]. The following primers were designed to amplify from the 3′ end of the transcript and used to detect phototransduction components in the resulting amplified cDNA obtained from single ipRGCs: Melanopsin (F: CTTTGCTGGATACTCGCACA; R: CAGGCACCTTGGGAGTCTTA), *Gnaq* (F: GTTCGAGTCCCCACTACAGG; R: GGTTCAGGTCCACGAACATT), *Gna11* (F: GTACCCGTTTGACCTGGAGA; R: AGGATGGTGTCCTTCACAGC), *Gna14* (F: CCATTCGACCTGGAAAACAT; R: CAGCAAACACAAAGCGGATA), *Gna15* (F: TGAGCGAGTATGACCAGTGC, R: CAGGTTGATCTCGTCCAGGT).

### Pupillometry

Pupil experiments were performed on unanesthetized mice that were restrained by hand. WT (16 animals), MKO (7 animals), *Gna11* KO (4 animals), *Gna14* KO (5 animals), *Gna15* KO (7 animals), *Gnaq; Gna11* DKO (9 animals), and *Gna11; Gna14* DKO (7 animals) were kept on a 12 hour∶12 hour light∶dark cycle and given at least 30 minutes to dark-adapt between stimulations. All experiments were performed during the animals' day (ZT2-10). The contralateral eye was stimulated with 474-nm LED light for 30–60 s. Neutral density filters were interposed in the light path to modulate light intensity and light intensity was measured using a photometer (Solar Light). High light indicates 1.4×10^16^ photons/cm^2^/sec, and low light indicates 7.3×10^13^ photons/cm^2^/sec.

### Wheel Running Behavior

Mice were placed in cages with a 4.5-inch running wheel, and their activity was monitored with VitalView software (Mini Mitter), and cages were changed at least every 2 weeks.

WT (14 animals), MKO (9 animals), *Gna15* KO (7 animals), *Gnaq; Gna11* DKO (8 animals), and *Gna11; Gna14* DKO (7 animals) mice were placed in 12∶12 LD for 17 days followed by constant darkness for 26 days. For phase-shifting experiments, each animal was exposed to a light pulse (500 lux; CT15) for 15 min, after being in constant dark for 18 days. Following constant darkness, all mice were also placed in constant light (500 lux) for 18 days.

### Quantification of circadian behavior

All free-running periods were calculated with ClockLab (Actimetrics) using the onsets of activity on days 10–17 of constant darkness similar to [3]. Phase shifts were calculated similar to [3] and described as follows: an onset for the day after the light pulse was predicted based on the onsets of the previous 7 days. Phase shifts were then determined based on the difference between the predicted onset and the shifted onset on the day after the light pulse. For all animals, the free-running period in constant light was measured with ClockLab (Actimetrics) using the onsets of activity on days 3–10 of constant light. Some animals (2 WT, 1 *Gna15* KO, 2 *Gnaq; Gna11* DKOs, 1 *Gna11; Gna14* DKO, and 1 MKO) reduced their activity so much that an accurate period could not be measured and they were thus excluded.

### Multielectrode Array Recordings

Multielectrode array recordings, light stimulation, and data analysis were performed as described in [11]. Briefly, retinas were dissected from P3 pups from WT and *Gna11*; *Gna14* DKO animals and mounted ganglion cell side down on the array. Retinas were superfused with Ames' Medium (Sigma) and synaptic blocker cocktail oxygenated with 95%/5% Oxygen/CO2. Synaptic blocker cocktail consisted of: 250 µM DL-2-amino-4-phosphonobutyrate; 10 µM 6,7-dinitroquinoxaline (DNQX, α-amino-3-hydroxy-5-methyl-4-isoxazolepropionic acid); 0.3 µM strychnine, 50 µM picrotoxin, and 10 nM (±)-epibatidine dihydrochloride. All reagents were purchased from Tocris (Ellesville, MO, USA). Spike sorting was performed using MCRack v 4.0.0 software (Multi Channel Systems)and analyzed offline with Offline Sorter v 2.8.6 software (Plexon Inc, Dallas, TX, USA).

### Q-RT-PCR

Retinas were dissected from WT, *Gna14* KO, *Gna15* KO, *Gnaq; Gna11* DKO, and *Gna11; Gna14* DKO (N = 3 mice for each; 2 retinas per RNA sample). RNA was extracted from the retinas using an RNeasy mini kit (Qiagen; cat# 74106), and reverse transcription was performed using a RETROscript kit (Life Technology; cat # AM1710) and random hexamer primers. Quantitative PCR on the resulting cDNA was performed with SYBR Green PCR Master Mix (Fermentas, cat# K0221), samples were analyzed in duplicate, and the levels were normalized to 18S RNA. The following primers were used: *Gnaq* (F: AATCATGTATTCCCACCTAGTCG; R: GGTTCAGGTCCACGAACATT), *Gna11* (F: TCCTGCACTCACACTTGGTC; R: GGGTTCAGGTCCACAAACAT), *Gna14* (F: TCACCTACCCCTGGTTTCTG; R: CCGCTTTGACATCTTGCTTT), *Gna15* (F: ACCTCGGTCATCCTCTTCCT, R: CGCATACATGTCCAAGATGAA), and 18S RNA (F: CGCCGCTAGAGGTGAAATTC; R: TTGGCAAATGCTTTCGCTC).
